# Insights to Improve Dietary Guidelines for Americans Communication and Policy

**DOI:** 10.3390/ijerph20186767

**Published:** 2023-09-15

**Authors:** Wendy Macias

**Affiliations:** Strategic Communication Department, Texas Christian University, Fort Worth, TX 76129, USA; w.macias@tcu.edu; Tel.: +1-817-257-4577

**Keywords:** health communication, American nutrition health, systems thinking

## Abstract

This study aims to tease out why the Dietary Guidelines for Americans (DGA) have largely failed to support positive attitudinal and behavioral dietary change in the U.S. over the past decade. Dervin’s sense-making methodology (SMM) is employed as a theoretical framework to postulate why DGA has not been more successful with its past communication approaches. A brief history of DGA is shared along with criticisms and literature discussing its potentially politicized nature. Thirteen semi-structured qualitative interviews with individual people and various experts (government, dietitians, communicators, and school-lunch administrators) reveal successes and failures and, ultimately, gaps in the communication process. Key themes emerged indicating the importance of mothers, coaches, and significant others, but rarely DGA, in American’s nutritional upbringing. Industry expert interviews exposed areas where competing systems may cancel out efforts and how too many people are looking at narrowly focused details without seeing the big picture. Systems thinking embedded in social change theories may be advantageous over traditional, less coordinated linear-thinking approaches to improve awareness and attitudes. A systems map is proposed to guide the process and bring key parties together to move beyond the contentious, only one winner mentality that has dominated in the past. In conclusion, this article adds qualitative insights to this area of inquiry and makes suggestions to improve organizational communication and policy.

## 1. Introduction

The Dietary Guidelines for Americans (DGA) are published every five years as a joint venture between the U.S. Departments of Agriculture (USDA) and Health and Human Services (HHS) since 1980. One of the goals is to inform citizens, organizations, and businesses and cultivate consumers who can take ownership of their health decisions and behaviors. This article examines their institutional organizational effectiveness through the lens of sense-making methodology (SMM) [1] to better understand how DGA may be successful, or not, in its goals. The author utilized a systems-thinking approach to help envision how change could more effectively be facilitated.

**Figure 1:**
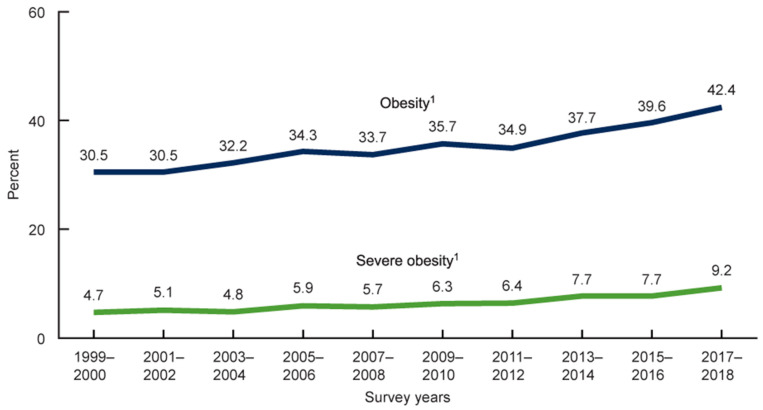
Rising obesity data for the US. (Public domain image reprinted from U.S. Department of Health & Human Services, Centers for Disease Control and Prevention National Center for Health Statistics.) ^1^ Significant linear trend. SOURCE: NCHS, National Health and Nutrition Examination Survey, 1999–2018 [2].

The fact that over 71% of U.S. adults are overweight or obese highlights the importance of this topic [2,3], and this trend has been increasing in recent years (see Figure 1).

“Although consumers say they are concerned about nutrition and are aware that eating a healthful diet is important for good health, this knowledge does not always translate into healthful diet behaviors or motivate behavior change” [4] (p. 678). The same qualitative study (with dieticians and consumers) provided suggestions on how to better communicate dietary guidance messages in ways that consumers may be more open to, such as removing guilt about food choices to mitigate an avoidance response and simplifying information to empower consumers to act. This need for empowerment may be related to a general movement in health communication, which has found the consumer increasingly taking an active role in their healthcare [5,6]. The reader should keep in mind that the consumer is the ultimate receiver of dietary information, but this study focused on how organizations, public policy, health professionals, and resulting interpersonal communication impact this process.

This study’s goals were to better illuminate why previous DGA research [7] found a lackluster impact of DGA on individual’s dietary knowledge, attitudes, and choices, as well as explore ways to improve Americans’ dietary health through public policy communication and practices. The data may help to inform a broader political and sociological question: what is more important, Americans or the American food industry? Although this study focused on dietary choices, the author acknowledges and supports the importance of physical activity for improving health.

### 1.1. Politics of Food Guidelines in the U.S.

Knowing the stated missions of the organizations involved in producing DGA is a good starting place to understand the underlying motivations. USDA states:

We provide leadership on food, agriculture, natural resources, rural development, nutrition, and related issues based on public policy, the best available science, and effective management. We have a vision to provide economic opportunity through innovation, helping rural America to thrive; to promote agriculture production that better nourishes Americans while also helping feed others throughout the world; and to preserve our Nation’s natural resources through conservation, restored forests, improved watersheds, and healthy private working lands [8].

HHS.gov states:

The mission of the U.S. Department of Health and Human Services (HHS) is to enhance the health and well-being of all Americans, by providing for effective health and human services and by fostering sound, sustained advances in the sciences underlying medicine, public health, and social services [9].

Another important website is created by the Office of Disease Prevention and Health Promotion (part of the HHS), and appears to act as a community-facing, education-focused communication vehicle where the DGA website resides. The Dietary Guidelines have a significant impact on nutrition in the United States, with the following aims:

- “Form the basis of Federal nutrition policy and programs
- Support nutrition education efforts
- Guide local, state, and national health promotion and disease prevention initiatives
- Inform various organizations and industries” [10], such as the food and beverage industry.

Given these missions, the dual purpose of DGA begins to emerge—they serve Americans and the American food industry. An opinion piece further outlined the challenges in communicating dietary guidelines to consumers (e.g., conflicting scientific studies, difficulties understanding probabilities), and that consumers may not be aware of the political nature of what is or is not included [11]. One book, *Food Politics* [12] took a deep dive into these politics, including things the public may not know about how dietary guidelines are determined, such as the political/business deals that take place behind the scenes. Nestle [12] shines a light on how much influence industries like meat, dairy, and grain may really have on what the DGA recommend Americans consume. News media are also writing about the frequent disconnect between nutrition science and policy [13,14,15].

### 1.2. Brief History of DGA Communications

**Figure 2:**
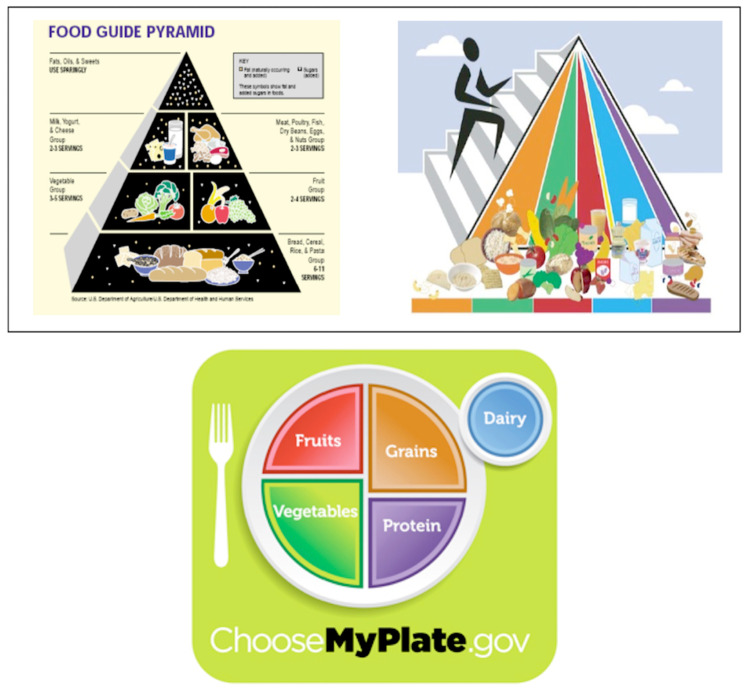
Graphic Representations of the DGA 2000 Food Guide Pyramid, DGA 2005 MyPyramid and 2010 MyPlate.gov. (Public domain images reprinted from health.gov, created by Office of Disease Prevention and Health Promotion, ODPHP, a division of U.S. Department of Health and Human Services).

Another aspect of the DGA further complicates how successful the USDA and HHS have been at disseminating dietary education to Americans. The contemporary history of the DGA over the past 15 years reveals several significant changes related to increasing physical activity, the consumption of healthier fats rather than less healthy alternatives, the importance of whole grains, increasing dairy consumption, and reducing sugar intake. To communicate these substantive changes to consumers, the graphic representation of the traditional Food Pyramid was modified and replaced with a new MyPyramid graphic that organized the food groups in vertical categories instead of horizontally. The different colors represent the food groups, and the person running beside the graph symbolizes the need to exercise. Figure 2 presents these three images and will serve as an organizer in the discussion of portions and the emphasized changes over the years (brief history available at https://www.choosemyplate.gov/brief-history-usda-food-guides) (accessed on 12 March 2023).

While, at times, the DGA has served as both a policy document and source of dietary information for the public, the past three editions (2010, 2015 and 2020) focused on offering guidance to professionals (healthcare workers, educators, etc.) who would share the information with patients and families using consumer-facing graphics. DGA 2020 specifies, “Dietary Guidelines is designed for policymakers and nutrition and health professionals to help all individuals and their families consume a healthy, nutritionally adequate diet” [16] (p. vii). Next, two theoretical frameworks will be discussed to help find a more effective way to understand and plan for healthy dietary changes and exercise recommendations.

### 1.3. Sense-Making Methodology

The sense-making methodology (SSM) provides a very useful approach to contextualizing and understanding how DGA works (or does not work) in communicating with Americans while considering the crowded media landscape of food promotions and the freedoms inherent to society [17]. SSM, designed to create and research public communication campaigns, could also be applied to message construction if DGA and its governing bodies, HHS and USDA, decided to develop more consumer-facing messaging like they did after the 2005 DGA release.

Several of the fundamental assumptions of SSM render it well-suited to our understanding of how and to what degree DGA impacts individuals. Specifically, it views communication’s impact on humans because of intrapersonal communication, where all participants have the freedom to interpret material based on individual experiences [16]. Public interest campaigns like DGA are inherently underfunded, if funded at all, especially in comparison to large food brands with multimillion yearly marketing budgets. SSM helps bring into perspective how DGA is not correlated with an increase in U.S. dietary health, but the opposite. It is easy to blame the audience for lack of compliance with the advice to eat healthier and exercise more when the challenges of everyday life have not fully been considered. How many Americans are truly motivated to make sense of the ever-changing landscape of dietary and physical activity research and keep up with guidelines that are not written with them in mind? Do our health and dietary systems provide the trickledown effect intended by DGA creators? Do children learn good eating and exercise patterns at school? Do adults receive proper dietary and exercise counseling to a large extent or only when in a health crisis?

### 1.4. Systems Thinking Theoretical Framework

Systems thinking for social change [1] provides a helpful framework for examining the DGA communications as a source of dietary nutrition information for Americans. Stroh [1] posits that a coordinated, systems approach to positive change involving complex social issues (homelessness, climate change, etc.) will be more likely to be successful than more linear traditional thinking for several reasons:

1. Linear thinking focuses on the symptoms but systems thinking looks for the underlying causes.
2. Linear thinking often employs temporary “quick fixes” as opposed to the long-term solutions of systems thinking.
3. Linear thinking may work well for very simple problems with limited factors, but systems thinking is better able to develop a nuanced approach to complex societal issues with many factors and intricate relationships.
4. Systems thinking integrates the change management theories needed to ensure long-term behavior and attitude change.

For these reasons, the author advocates for systems thinking to be applied by the government, health professionals, and nonprofit agencies involved with DGAs to provide a coordinated communication approach to help Americans make the necessary changes in their nutrition attitudes and behaviors to flatten the curve on the obesity pandemic.

Public health and health communication research have applied and advocated for systems thinking and helped to inform this research by supporting the idea that advances in public health require working together across various disciplines/departments and finding better ways to communicate and understand complex problems [18,19,20,21]. Systems thinking helps make implicit models explicit so they can be more accurately mapped to find viable means to enact positive change [21].

Based on this review, the following research questions guided the study:

(RQ1) Using the SSM framework, how do the context (power structures, organizational systems, and culture) and situational factors (history, experience, barriers, habits, and skills) inform our understanding of DGAs’ impact and individuals’ dietary knowledge or result in gaps (questions, confusions, or stress)?

(RQ2) Is the current structure for compiling and communicating DGA effective? How might it be improved by employing systems thinking?

## 2. Materials and Methods

To complement the scarce and predominantly quantitative survey data on DGA [7] and attempt to develop a more complete contextual picture, 13 in-depth interviews were conducted with women (*n* = 8) and men (*n* = 5) of various ages (21–74), occupations (college students, government USDA program coordinator, lawyer, process specialist, program coordinator, dietitians, communicators, teachers, and school-lunch administrators), and demographic backgrounds (e.g., white, black, Hispanic, and Asian race; including all major geographic regions in the U.S.) during the summer of 2012, fall 2017 and summer 2022. The research was approved by the author’s IRB (#M-83) and all participants gave fully informed consent. This research design did not attempt to oversample women; rather they naturally emerged as the most highly involved in health and nutrition, and most willing to participate. Women were also more eager to talk about the topic and share their dietary knowledge journey. Scant research has directly investigated this relationship but research does support the idea that women are more involved in the health of the family, broadly defined [22].

A purposive sampling technique attempted to provide the broadest perspective possible, including people representing consumers ranging from a health zealot to an “average” American, as well as professionals including a registered dietitian, nutrition-related administrators, and public-school teachers. This sampling technique aimed to illuminate how DGA is known and understood by consumers as a result of the HHS and USDA’s indirect communication model. Semi-structured interviews lasted 30–60 min and asked (1) (for individual consumers) how they learned about nutrition over their lifetime and what role, if any, DGA played, and (2) (for professionals) how they formally or informally communicate DGA and to envision how the average consumer may encounter DGA.

Given the qualitative nature of this study, the author provides a personal background to let the reader judge any bias that may be present, despite every effort to let the data tell the story. As a health communication researcher, the author is motivated to help consumers be healthier, make informed decisions, and lead a full life, while understanding that not all individuals may define those concepts the same way. Even so, the author does believe Americans should be able to trust the information provided by their government—yes, that may sound naïve, and the reader should know the author’s natural disposition is to trust. The author is a scientist who loves digging to find the truth or working towards that goal. The reader can judge the truth for themselves.

## 3. Results

### 3.1. RQ1: Context Informs DGAs Impact and Individual’s Dietary Knowledge

Several key insights and themes emerged from the data that augment our understanding of American’s dietary knowledge, including DGA, by adding context and individual detail.

#### 3.1.1. Mothers Are Key Influencers

In most consumer interviews, mothers were mentioned first and most often when participants were asked how they formed their dietary and nutrition knowledge throughout their lives.

- “I always remember my mom packing my lunch with both a fruit and a veggie, insisting I eat both before the chips.”
- “My mom would be sure to include a vegetable with our family dinner, even if it was a piece of lettuce rolled up.”
- “My dad helped make meals a lot, but mom was always the one advocating for healthier options at home and when eating out.”
- “Thinking back now, I can see what a big impact my mom had on my ideas of healthy eating and the importance of exercise.”
- “Mom also would never let us believe we could do fad diets, like drink grapefruit juice all day.”

#### 3.1.2. Coaches’ Impact on Athletes

About half of the interviewees mentioned coaches as the second key influencer in nutrition knowledge. Obviously, this only applied to those who participated in sports, but the influence was strong, nonetheless. Several of the women described this as a pivotal point in their personal nutrition biography. It was described as a time of heightened awareness of the effect that nutrition had on their body and an increased involvement in the issue. For several of these women, this involvement has persisted or even increased 10–15 years later. “Certain magazines, like *Runner’s World* or *Cooking Light*, became trusted sources of information because they were leading by example or cited research studies that were fascinating”.

#### 3.1.3. Significant Others Importance Later in Life

Another theme that emerged was the importance of significant others in realizing a healthy change was needed. For one young woman, the impetus was wanting to help her father be healthier and improve his diet, and for another, her husband was the catalyst. While the women were primarily motivated to improve their significant other’s health, their own nutrition knowledge and behaviors improved as well. Another said he “initially learned some through school and the food pyramid…growing up we just ate what we had access to. I have learned more in my adult life from my husband”.

#### 3.1.4. Self-Taught Nutritionists

Several participants shared that they primarily taught themselves, and one participant expressed this particularly well—“I was aware of the U.S. pyramid and circular thing, but it really didn’t have much impact on me. I developed my own personal model and try to eat ‘good things’ in abundance and ‘bad things’ in moderation while keeping genetics leanings in mind” (i.e., diabetes and heart disease). He learned about nutrition indirectly because his family had a large garden and primarily ate a vegetable-based diet. It was not until later in life that he sought out more information and became self-taught through cookbooks, books, and online sources about nutrition and diet.

#### 3.1.5. Anti-Theme: Absence of DGA

DGA was absent from all but one discussion and only emerged after direct prompting—“I think I remember learning about that in school.” Several could offer vague details about “food groups” and descriptions of “the plate thing” or “food pyramid.” However, one participant had a more extreme view—“I thought dietary guidelines were heavily influenced by various lobbyists and not rigorous, independent research. The food pyramid was something that a bunch of rich, white men developed to help the economy by getting people to eat all this food”.

#### 3.1.6. Industry’s Focus on the Trees, without Seeing the Forest

Several interviews with industry professionals across multiple sectors indicated a lack of a coordinated approach with a clear goal in mind when communicating the DGA. Each sector is focused on their piece of the puzzle, but there is little to no coordination across or between units. This is common with traditional linear thinking as opposed to system thinking [1]. When collaborations are not well-coordinated, thinking and activities tend not to aim towards the same goal, miss the big picture or do not see how they may inadvertently make their own, or other, efforts less effective. For example, the USDS and HHS work together to release the DGA, alternating as lead (HHS was lead in 2015, USDA in 2010, etc.), and each of them has their own lane, to some extent, with the HHS being the nutrition expert and the USDA representing the food perspective.

To further complicate this from an organizational and communication perspective, regional and local entities (e.g., state agricultural departments and school districts) have their own mandates and missions. While all these may be working to help Americans be healthy and make food available to individuals, a lack of coordination hinders their ability to head towards the same goal.

### 3.2. RQ2: DGA’s Communication Structure, Effectiveness, and Improvement

Several key insights and themes emerged from the data that augment our understanding of American’s dietary knowledge, including DGA, by adding context and individual detail.

#### Health Professionals’ Attitudes Towards DGA

Several interviews with professors of nutrition, nursing, and kinesiology, as well as a registered dietician, indicated that the MyPlate graphic was often helpful in explaining healthy eating to outreach clients they worked with: “It was easy to explain, understand, and utilize.” They liked that it showed both types of food and portion sizes. They like it because it is simple.

The dietician’s viewpoint about the graphic’s changing over the years, from a food pyramid to MyPyramid to MyPlate, was that the DGA and graphics are not separate entities but iterations along a continuum that can continue to work together. She continues to use them all, especially if people are still familiar with them all. She finds it helpful to show the development of nutrition knowledge and the details of good nutrition that affects people’s everyday lives.

Nutrition and health knowledge is ever-evolving by design and necessity. The DGA was created to help consolidate that knowledge into a more comprehensible package for Americans. While the quantitative research showed significant awareness of the 2005 MyPyramid and decreasing awareness of subsequent iterations [7], the qualitative interviews indicated that personal influences were more impactful and memorable. This disparity could indicate that individuals rely more on people close to them than on a government report to inform them about nutrition, aligning with the assumptions of SMM. However, after the 2005 DGA, there was less consumer-facing communication and more of a two-step information flow through health professionals.

## 4. Discussion

Given the extreme nature of the obesity problem in the U.S., these findings do not present a very positive picture of Americans’ awareness of a major source of nutritional and exercise information and guidelines (DGA2005, 2010, 2015; MyPyramid/MyPlate) or America’s ability to communicate to individuals effectively. MyPyramid was actively promoted through advertising (outdoor, TV, online and print), as well as posters to schools, government agencies (i.e., WIC) and medical offices. It is possible that these messages were either ignored or got lost in the clutter of other health messages and nutrition-focused advertising. Another factor may be that communication is the first step in raising awareness of the lifestyle and dietary changes needed to be healthy; however, since this requires significant behavioral changes for many Americans, more than communication is needed to support positive change. As previous research has noted, awareness of the DGA does not always mean individuals are using them to make dietary and activity choices or improvements [7].

### 4.1. Interpersonal Impacts

Over the years this study was conducted, there was a definite shift from mass media, with social media and interpersonal influences becoming more prominent. One way the interpersonal influence was displayed was through athletes taking greater control of their health, making their own decisions, gaining independence from their parents, and being more keenly aware of the impact that food has on their bodies because of their involvement in sport.

### 4.2. Dietary Guidelines and Systems Thinking

Communication regarding healthy eating in the U.S. quickly becomes a labyrinth for the consumer. Brands are trying to persuade consumers to buy their products. Various federal (FDA, USDA, HHS, etc.), local, and regional government groups are communicating their dietary missions. A myriad of family, friends and health providers are mining available information and passing it along to their personal and professional networks as necessary. As is common in our society, this creates a lot of information, and sometimes misinformation, that most often lacks coordination and is difficult for busy consumers to filter through and decide what to act on. The DGA, and nutrition communication in general, would benefit from the systems approach that has been successfully used to tackle other complex social issues like homelessness and education inequality [1].

**Figure 3:**
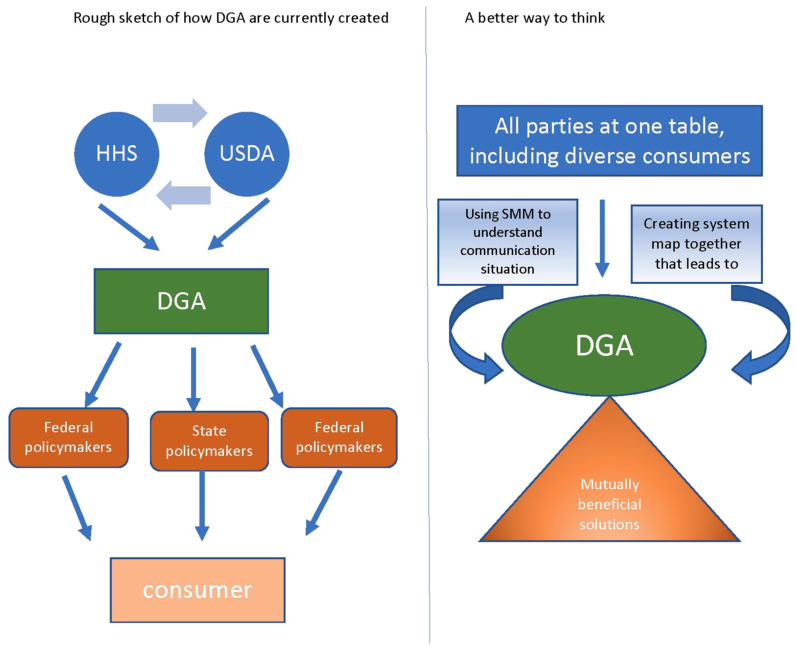
Linear versus systems thinking.

This study suggests bringing together all interested and affected parties, including the government, individuals, health professionals, communicators, and food producers, to learn from each other and see how the system is currently working, or not working. Developing a systems map (see Figure 3) can help those involved to understand each other’s goals and come to an agreement on how the system needs to change, thus leading to positive, healthy change for all Americans, including individuals, businesses, communicators, and health providers. Here is a first attempt at a systems map of America’s dietary information culture.

As a map of this evolving system, all constituents should work towards understanding the system, which would open the way for system-wide change. Currently, business, government and consumers are in a power struggle, assuming that a viable solution would mean a loss of power or giving something up. Currently, there are three systems archetypes [1] that may be hindering the chances of success: (1) accidental adversaries (various parties are more likely to compete than cooperate), (2) drifting goals (what helped to educate in a 1980 media landscape is no longer effective), and (3) competing goals (without working together, competing goals lead to nothing getting accomplished).

Conventional linear thinking is faulty for several reasons, as pointed out in Stroh’s 2015 book [1]:

1. The causes of problems are obvious and easy to find.
2. Someone else is to blame and must be the one to change.
3. Policies that can achieve short-term success will also lead to long-term success.
4. Impacting the whole situation can be achieved through optimizing the constituent parts.
5. Aggressively attacking various parts of a problem is better than coordinating over time in a strategic manner.

## 5. Conclusions

Seeing where and to what degree Americans have learned about the DGA is a crucial step in envisioning how future DGA can more effectively be disseminated. While there may be advantages of the MyPlate graphic as a tool to help Americans, the DGA cannot continue to change its consumer-facing graphics every five or ten years in such a substantial way and expect the average consumer to fully benefit and make positive changes. This is not meant to say that the DGA should not continue to incorporate research on the best dietary recommendations; rather, there needs to be a consistent structure to incorporate new information. This could be similar to a brand’s campaign, which may change over the years to ensure it remains effective and fresh in the consumer’s mind. This is an important first step in helping Americans make healthy changes to their diet, exercise, and lifestyle. Public health messages are far too often are viewed as one-shot inoculations rather than brand-building campaigns that build on previous knowledge. For example, the addition of exercise to the DGA in 2005 was not included in the MyPlate graphic and may not remain a focus for the consumer. In addition, a more coordinated systems approach would help ensure that these efforts are making a difference and leading to a healthier American population.

Limitations exist for any study. The reader is encouraged to consider the key limitations of this study, which include the sample size; however, research has found that qualitative saturation can be reached in sample sizes of as small as 9–17 [23]. The sample was purposive, and not meant to be representative, and did skew female. The data and discussion should be considered one way, not the only way, to understand the topic.

## Data Availability

Data is unavailable due to privacy and ethical restrictions.
